# WAVOS: a MATLAB toolkit for wavelet analysis and visualization of oscillatory systems

**DOI:** 10.1186/1756-0500-5-163

**Published:** 2012-03-26

**Authors:** Richard Harang, Guillaume Bonnet, Linda R Petzold

**Affiliations:** 1 Department of Computer Science, University of California, Santa Barbara, CA, USA; 2 Department of Statistics and Applied Probability, University of California, Santa Barbara, CA, and Google Inc, Mountain View, CA, USA

## Abstract

**Background:**

Wavelets have proven to be a powerful technique for the analysis of periodic data, such as those that arise in the analysis of circadian oscillators. While many implementations of both continuous and discrete wavelet transforms are available, we are aware of no software that has been designed with the nontechnical end-user in mind. By developing a toolkit that makes these analyses accessible to end users without significant programming experience, we hope to promote the more widespread use of wavelet analysis.

**Findings:**

We have developed the WAVOS toolkit for wavelet analysis and visualization of oscillatory systems. WAVOS features both the continuous (Morlet) and discrete (Daubechies) wavelet transforms, with a simple, user-friendly graphical user interface within MATLAB. The interface allows for data to be imported from a number of standard file formats, visualized, processed and analyzed, and exported without use of the command line. Our work has been motivated by the challenges of circadian data, thus default settings appropriate to the analysis of such data have been pre-selected in order to minimize the need for fine-tuning. The toolkit is flexible enough to deal with a wide range of oscillatory signals, however, and may be used in more general contexts.

**Conclusions:**

We have presented WAVOS: a comprehensive wavelet-based MATLAB toolkit that allows for easy visualization, exploration, and analysis of oscillatory data. WAVOS includes both the Morlet continuous wavelet transform and the Daubechies discrete wavelet transform. We have illustrated the use of WAVOS, and demonstrated its utility for the analysis of circadian data on both bioluminesence and wheel-running data. WAVOS is freely available at http://sourceforge.net/projects/wavos/files/

## Background

Many real-world sources of data display suggestively periodic behavior, but with time-varying period, amplitude, or mean. This variation can lead to inaccurate results when the data is analyzed with standard Fourier techniques, as Fourier analysis assumes stationarity of the signal and its basis functions are unbounded in time [1]. Wavelets, in contrast, are localized in both time and frequency. This in turn localizes the analysis, allowing the changes insignal properties to be tracked over time [2].

Wavelet analysis has proven to be invaluable in many problem domains, including ecological cycles [3], sunspot cycles [4], circadian cycles [5,6], nfradian cycles associated with gene transcripts [5], blood-flow dynamics [7], and ECG signals [8-10]. Our work todate has focused on circadian data. This data typically displays many of the features that render a signal difficult to analyze via Fourier techniques, including changes in period length, sharp transients, and phase shifts [6,11], as well as experimental artifacts such as loss of amplitude, shifting means [12], and "shot noise" when bioluminescence experiments are considered [13]. These features may require significant pre-processing of the data before analysis [14]. Recognizing the potential of wavelet methods for analysis of circadian data, Price et al. [5] developed the WAVECLOCK software, implementing the Morlet CWT in the R statistical programming environment. The WAVECLOCK software has enabled and inspired several investigations [15,16], but requires familiarity with the R statistical programming language and command-line interface. Other wavelet implementations (e.g. WaveLab [17] or the MATLAB Wavelet Toolkit) exist, but are either general-purpose packages that assume significant familiarity with wavelets, proprietary software, or both.

The wavelet transform, in both continuous (Morlet) and discrete (Daubechies) versions, offers a set of tools for the analysis of nonstationary oscillators that can avoid many of the issues associated with techniques that assume stationarity. The Morlet continuous wavelet transform can nonparametricallydenoise, detrend, and analyze the local frequency content of a signal in a single operation [1,15]. Application of the CWT enables such analyses as estimating the evolution of the period and phase of a signal across time, locating peaks and troughs even in the presence of large amounts of noise, tracking the evolution of the amplitude across time, and identifying multiple simultaneous oscillators. The discrete wavelet transform (DWT) enables multi-scale analysis of a signal using a sequence of compactly supported filters that decompose the signal into a set of component frequency bands [18]. While it yields less precise frequency estimates than the CWT, it enables direct statistical significance testing of different frequency bands [19], and has better time localization properties than the CWT, allowing for more efficient removal of transients that are strictly time-limited. It also has reconstruction properties that are capable of preserving the mean of the signal [18]. Thus the DWT can be used as a pre-processing step for other, non-wavelet analyses. Readers interested in learning more about the continuous wavelet transform are referred to [2]; for further information on discrete wavelets, see [19].

**Figure 1:**
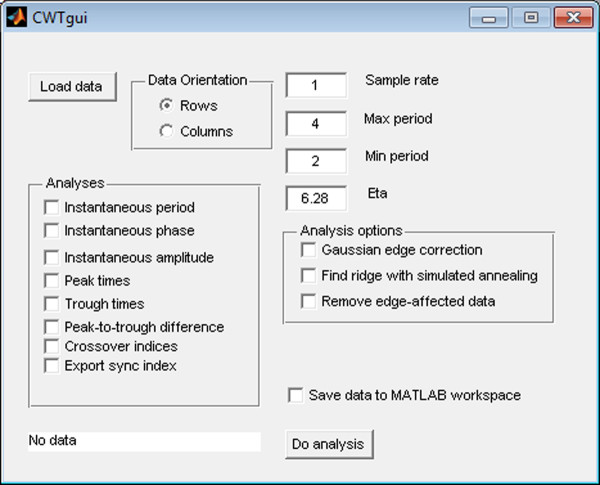
**CWTgui main interface**. The main user interface of CWTgui, containing controls to load the data, select analysis parameters, and select the output type and location. CWTgui performs batch analysis of a dataset using the Morlet CWT and returns the results as either a MATLAB environment variable, or saved to a file.

**Figure 2:**
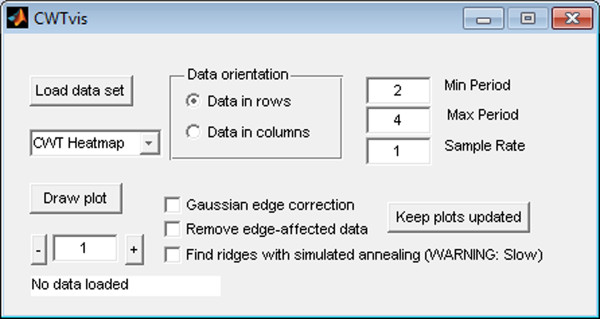
**CWTvis main interface**. The main user interface of CWTvis, containing controls to load the data, select analysis parameters, and explore various plotting and output options. CWTvis allows the user to visualize the output of wavelet operations on individual data traces before beginning a batch analysis using CWTgui.

**Figure 3:**
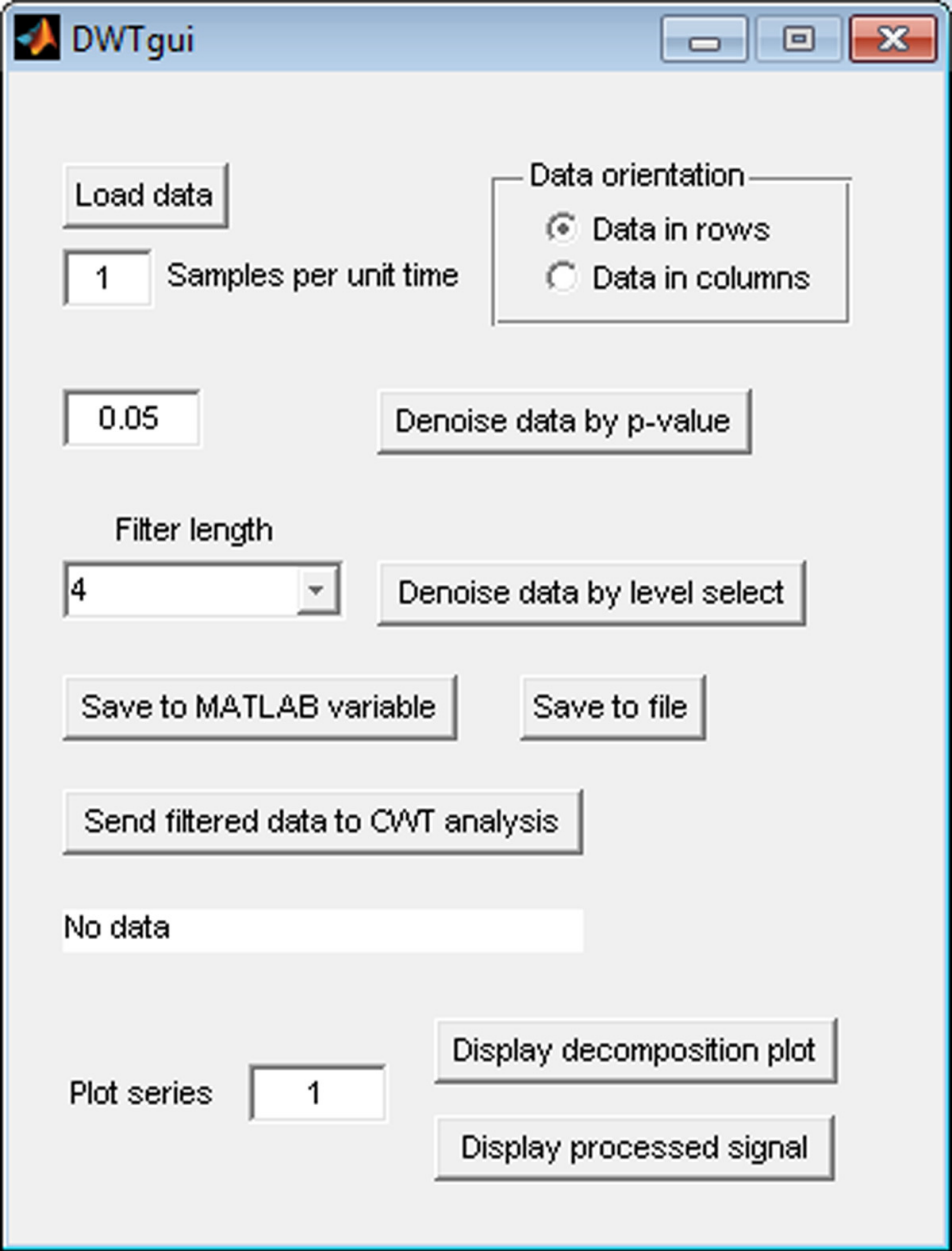
**DWTgui main interface**. The main user interface of DWTgui.

We have designed WAVOS to be a comprehensive, user-friendly toolbox for wavelet analysis and visualization of oscillatory signals that makes beginning-to-end wavelet processing and analysis accessible to users with a minimum of expertise in mathematics or computing. We present several analyses via a graphical user interface as illustrated in Figures 1, 2, 3. In the following, we discuss the capabilities and use of WAVOS and some results for circadian systems that we have recently obtained using this software. While the focus of this article is on circadian data, and the preset options are targeted towards such analysis (as described in the sequel), the WAVOS GUI and included functions may be readily used to analyze data of any periodicity by adjusting the minimum and maximum wavelength parameters.

### Implementation

WAVOS is implemented in MATLAB to allow for easy modification. The convolutions required for both the discrete and continuous wavelet transforms are performed in the Fourier domain. Several options are provided for managing the implicit periodization and corresponding boundary effects that this produces in the CWT, including zero-padding, edge-reweighting, and truncation of affected CWT coefficients (as in [5]).

For the discrete wavelet transform, the standard decimated transform may cause localization issues for particularly sharp transients, depending upon the place at which the signal is split during decimation. The transient may be completely contained in a particular coefficient of the transform, or it may be split between two adjacent coefficients. This has the undesirable effect of potentially allowing two different rotations of identical data to produce two different sets of DWT coefficients [19]. To avoid this issue, we use a redundant version of the DWT known in various contexts as the "undecimated", "shift-invariant", or "maximum-overlap" DWT [19]. This transform is invariant to rotation of the data. Statistical significance testing of the levels of the DWT decomposition is performed using a Chi-squared test, under the assumption of additive Gaussian noise as described in [19].

The default parameters in the GUI are geared towards analysis of circadian signals. In particular, the minimum and maximum periods default to 6 and 48, respectively, with a default sampling rate of 1. These presets assume hourly samples of data with periods of interest in the above range. Both the sampling rate and the periods of interest may be adjusted independently of each other. The tool will not attempt to analyse periodicities that exceed the minimum or maximum possible due to numberof samples and sampling rate, and will emit a warning message with the actual bounds used if such an analysis is attempted. For DWT denoising, a default p-value of 0.05 is used, as this is a generally accepted standard for statistical significance. Users may also select different levels of the decomposition by hand, in order to focus on periodicities of the data that interest them.

Due to the native MATLAB implementation, long time series (on the order of 10,000 or more observations) may result in analyses requiring several minutes per time series to complete. This may be mitigated by reducing the range of periods to be analyzed in the CWT, or down-sampling the series to a shorter one with a smaller number of samples per unit time. Data sets containing a large number of time series with a large number of samples may exhaust MATLAB's working memory, causing an error; this will depend strongly on the computer being used. Due to MATLAB's representation of data in matrix form, all time series in a single data set to be processed as a batch must contain an equal number of observations. Time series with differing numbers of observations may be processed and analyzed separately.

### Usage

The CWT and DWT have different features that incline them toward different uses. The Morlet CWT is most useful for investigating local properties of a signal: its detailed decomposition of time and frequency allows for very close tracking of several statistics of interest, however it cannot efficiently reconstruct a signal from individual components. The DWT fills a complementary role; while its time-frequency decomposition is very coarse, it is capable of reconstruction of a signal and also permits statistical testing. This allows the DWT to be used for many familiar signal-processing tasks, such as denoising and detrending, that are difficult to accomplish with the CWT.

**Figure 4:**
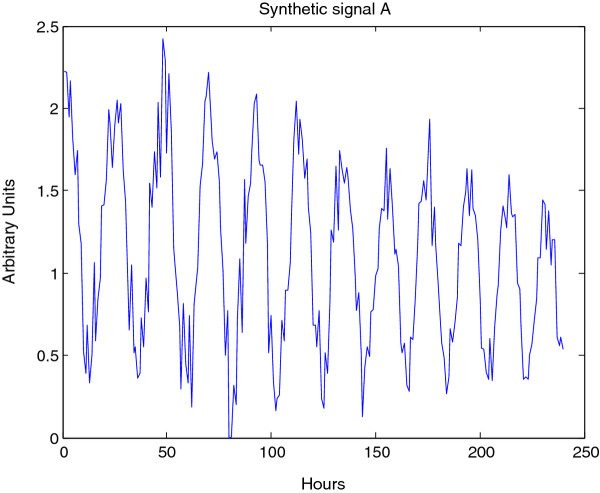
**Synthetic signal A**. Synthetic signal A; decreasing period from 24 to 18 hours, stable baseline, decaying amplitude. Shifting periods are not effectively detectable by the Fourier transform, and will lead to poor estimates of the period. In contrast, analysis by the CWTyields local estimates of the period.

**Figure 5:**
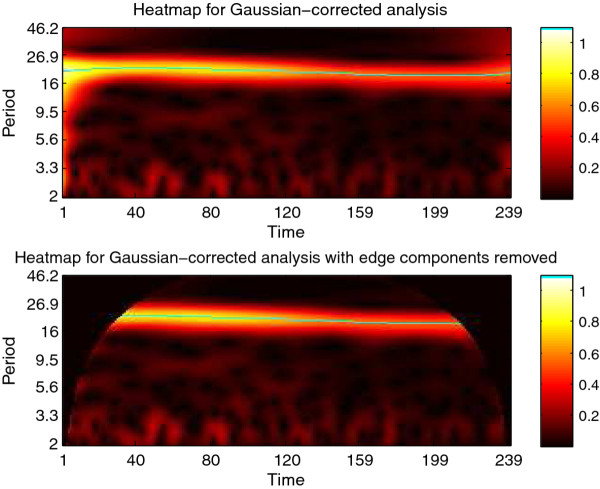
**CWTheatmap of synthetic signal A**. The first step of analysis of synthetic signal A (Figure 4) with the CWT: generating the CWT heatmap. The upper panel shows the complete CWT heatmap, the lower panel shows the CWT heatmap with all edge-influenced coefficients removed. The ridge is overlaid in green.

**Figure 6:**
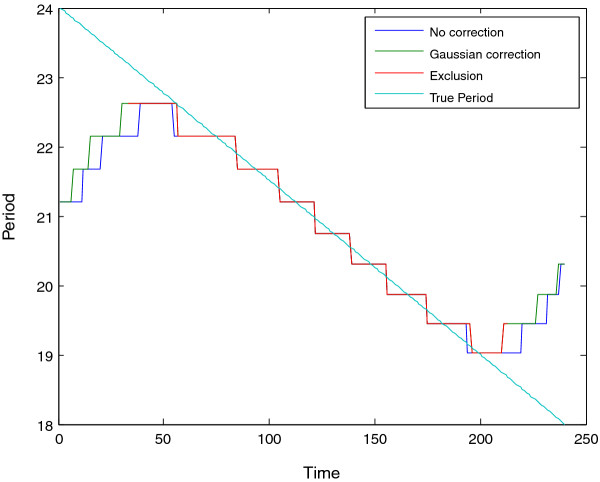
**Period estimates for synthetic signal A**. Estimated period of synthetic signal A (Figure 4), showing three different edge correction options. These estimates are derived from the ridge of the heatmap as shown in Figure 5. The true period is overlaid in green. This data may be plotted via CWTvis and exported to either an external file or a MATLAB variable with CWTgui.

**Figure 7:**
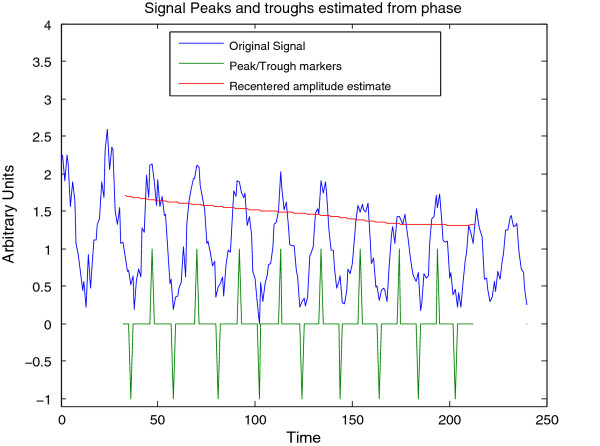
**Feature extraction for synthetic signal A**. Estimated peak, troughs, and instantaneous amplitude of synthetic signal A (Figure 4) estimated via the CWT; this plot may be visualized in CWTvis and exported to either an external file or a MATLAB variable with CWTgui.

The Morlet CWT may be viewed as a modification of the windowed Fourier transform (WFT) that scales the size of the window to a constant number of wavelengths for any analysis frequency [2], resulting in good frequency detection across a wider range of periods than many other methods. For example, Figure 4 displays a synthetic signal with a period that is shifting from 24 to 18 hours in length. Figure 5 shows the CWT heatmap of that signal with Gaussian edge correction applied and the edge-affected coefficients both included and excluded (all options that can be set within the WAVOS CWT module, see Figures 1 and 2). Note that the ridge of the heatmap (highlighted in green) tracks the changing period of oscillation. The changing period appears in more detail in Figure 6, where the instantaneous period is estimated using several different options within WAVOS. The Fourier-transform-like nature of the Morlet CWT allows for estimation of phase but localizes the signal, thus improving the accuracy of locating associated features such as the signal peak and trough. Figure 7 shows the peak and trough points of the signal, as well as the instantaneous amplitude measurements, obtained from the phase along the ridge identified in Figure 5.

**Figure 8:**
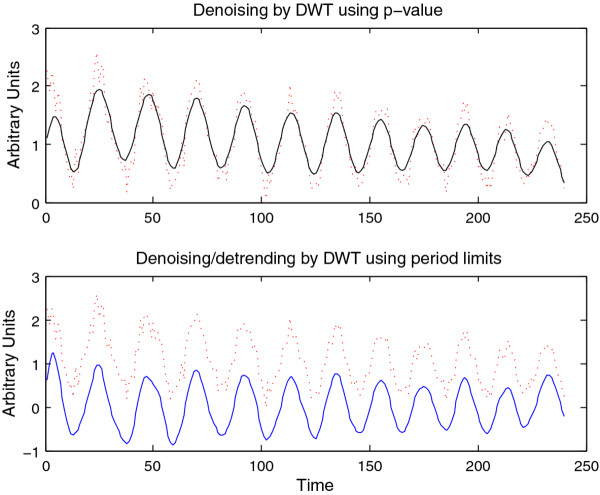
**Denoising/detrending of synthetic signal A**. Denoising/detrending of the synthetic signal A (Figure 4) using the DWT. The upper panel displays the output of the DWT processing of signal A on the basis of p-value, retaining only those coefficients with a p-value of less than 0.05. The lower panel displays the output of the DWT processing of signal A by hard-thresholding period boundaries. Note that p-value based processing typically retains a non-zero mean as well as moving baselines, since these tend to be statistically significant.

**Figure 9:**
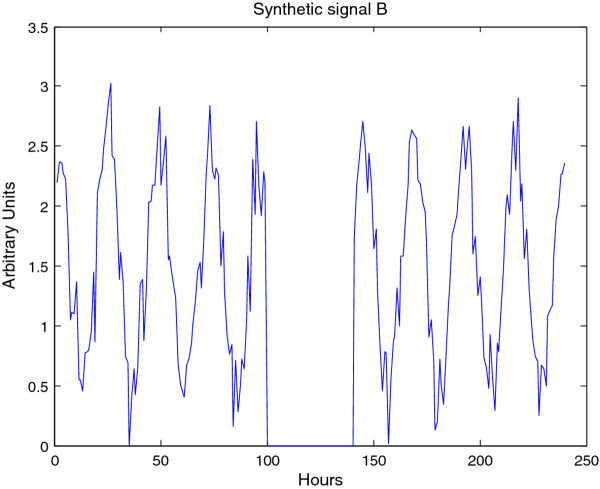
**Synthetic signal B**. Synthetic signal B; stable period with censored middle. The gap causes Fourier methods substantial difficulty.

**Figure 10:**
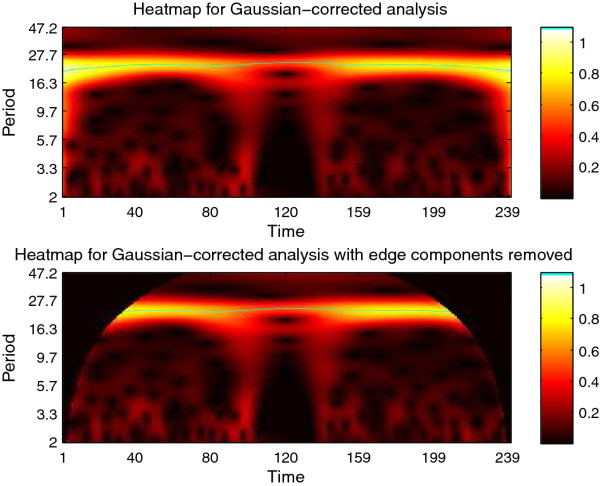
**CWTheatmap of synthetic signal B**. The first step of analysis of synthetic signal B (Figure 9) with the CWT: generating the CWT heatmap. The upper panel shows complete CWT heatmap. The lower panel shows the CWT heatmap with all edge-influenced coefficients removed. The ridge is overlaid in green. Note that a small distortion is created by the missing data, however the CWT is capable of interpolating over this range.

**Figure 11:**
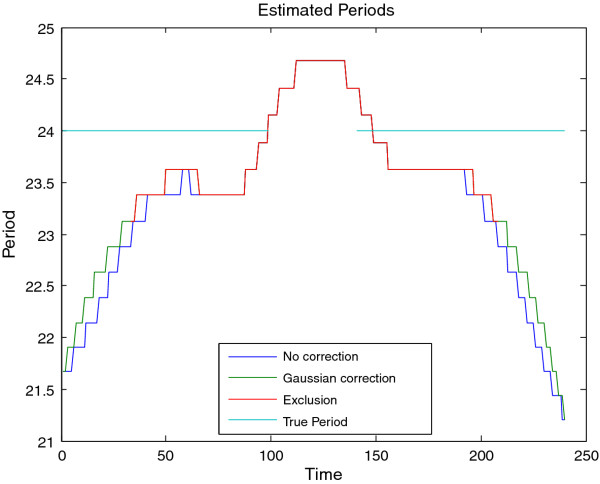
**Period estimates for synthetic signal B**. Estimated period of synthetic signal B (Figure 9), showing three different edge correction options. These estimates are derived from the ridge of the heatmap as shown in Figure 10. The true period is overlaid in green. This data may be plotted via CWTvis and exported to either an external file or a MATLAB variable with CWTgui. As in Figure 10, the period estimate is successfully interpolated across the missing data, albeit with some distortion.

**Figure 12:**
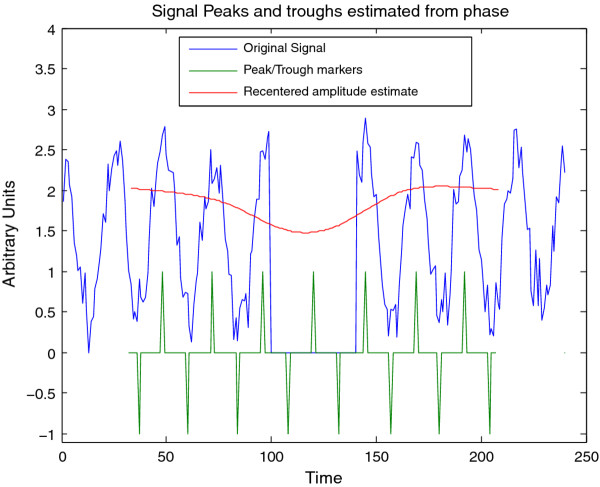
**Feature extraction for synthetic signal B**. Estimated peak, troughs, and instantaneous amplitude of synthetic signal B (Figure 9) estimated via the CWT; this plot may be visualized in CWTvis and exported to either an external file or a MATLAB variable with CWTgui. The amplitude estimate suffers somewhat from the missing data, however the peak-trough estimates are successfullyinterpolated.

**Figure 13:**
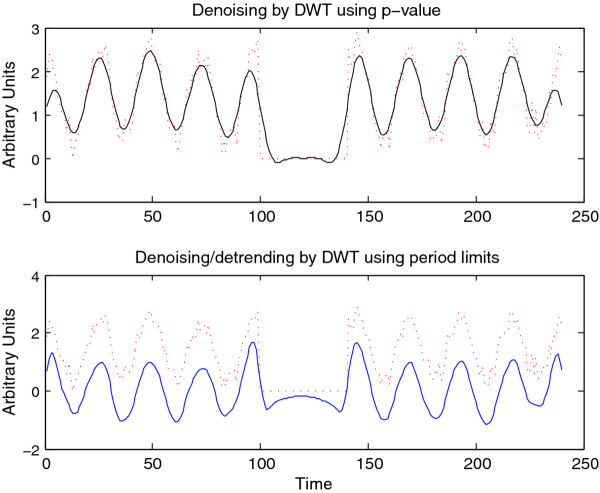
**Denoising/detrending of synthetic signal B**. Denoising/detrending of the synthetic signal B (figure 9) using the DWT. The upper panel displays the output of the DWT processing of signal B on the basis of p-value, only retaining those coefficients with a p-value of less than 0.05. The lower panel displays the output of the DWT processing of signal A by hard-thresholding period boundaries. Note that p-value based processing typically retains a non-zero mean as well as moving baselines, since these do tend to be statistically significant. Hard-thresholding to remove baselines can lead to some distortionof missing data as seen in the lower panel.

The CWT is excellent for examining local features of a signal. On the other hand, it is not well-suited for signal processing tasks such as decomposing the signal into different wavelengths, discarding or shrinking some coefficients related to particularwavelengths, and reconstructing the signal. Fortunately, the Discrete Wavelet Transform is well-suited for these tasks. Figure 8 illustrates the use of the DWT in two different modes provided by WAVOS (see Figure 3) to process the signal and remove either statistically insignificant noise, or portions of the signal outside of a particular range of frequencies. An analysis of a signal with missing data is provided in Figures 9, 10, 11, 12, 13; these figures illustrate the robustness of the CWT and DWT to significant amounts of missing data. All figures were generated using WAVOS.

Using the Morlet CWT in WAVOS has enabled us to analyze the distributional properties of the period of rat SCN cells based on PER2::LUC bioluminescence data [6]. Where previous studies had classified cells as either arrhythmic or circadian, our wavelet analysis revealed that individual cells, when removed from network interactions, intermittently express circadian and/or longer infradian periods. Results from our stochastic model suggest that the uncoupled cells may beswitching between two ocsillatory mechanism: the indirect negative feedback of protein complex PER-CRY on the expression of Per and Cry genes, and the negative feedback of CLOCK-BMAL1 on the expression of the Bmal1 gene.

**Figure 14:**
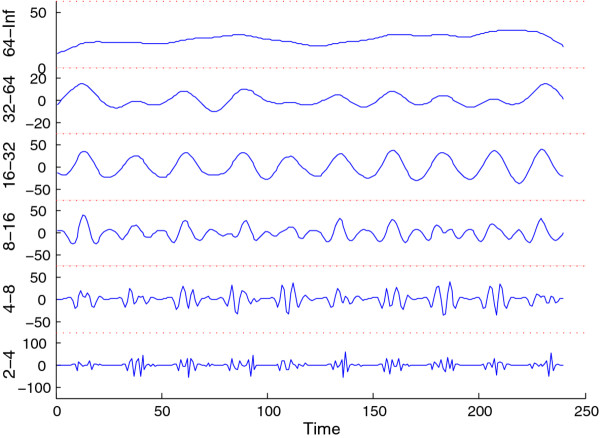
**DWT analysis of wheel-running data**. The DWT decomposition plot of 10 days of wheel-running data from a wild-type mouse in a 12:12 light schedule (generously provided by the Herzog lab).

**Figure 15:**
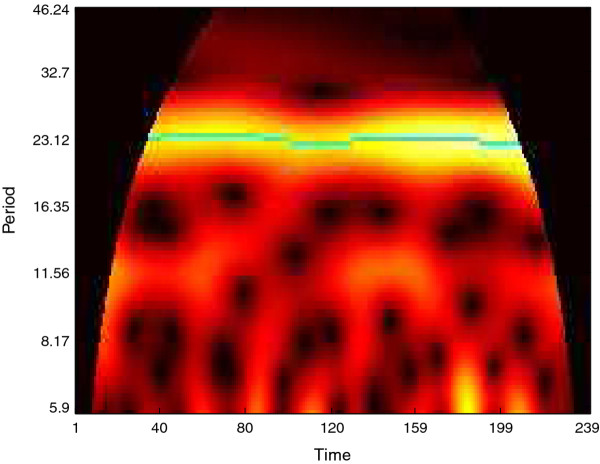
**CWTheatmap of wheel-running data**. The CWT heatmap of the data of Figure 14. Note that the period is relatively stable over the course of the data and that a single ridge is clearly visible, as well as some brief ultradian periodicities.

**Figure 16:**
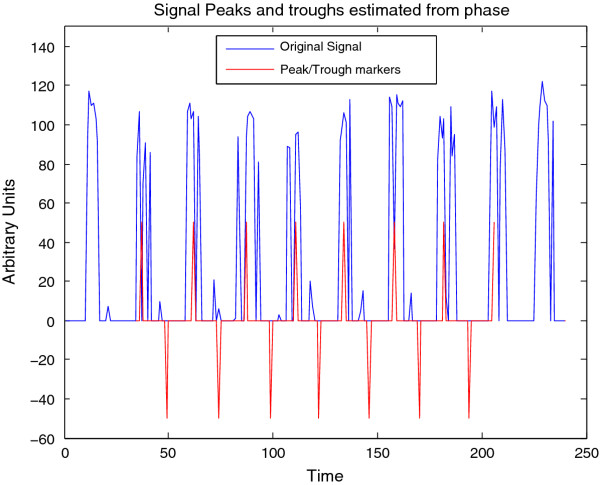
**Peak and trough markers for wheel-running data inferred by CWT analysis**. The ridge and phase information as displayed in Figure 15 were used to infer peak and trough phase markers for the signal, here overlaid upon the original data. Note that despite the distinctly non-sinusoidal pattern of behavior, the CWT is capableof automatically inferring peak activity and trough activity from the provided data.

As another example, we have also used WAVOS to analyze wheel-running data for wild-type mice in a 12:12 light:dark cycle (unpublished data, courtesy of the Herzog lab). The data contains wheel revolutions per minute sampled every 15 minutes for 10 days. Figure 14 shows the DWT decomposition plot; the main periodicity of roughly 24 hours is clearly visible in the 16-32 hour band, and the noise associated with waking behavior is visible in the highest frequency/shortest period bands at the bottom. The same data is analyzed in Figure 15 via CWT; the circadian periodicity of 24 hours is clearly visible, highlighted via the ridge, along with intermittent ultradian rhythms. Edge effects have been removed (black masking) to avoid potential artifacts in the analysis. Finally, Figure 16 shows peak and trough phase markers inferred automatically from the CWT (green) overlaid on the original data (blue). As above, edge-effect masking has been performed. Despite the noisy and non-sinusoidal nature of the data, the CWT accurately selects the appropriate phase markers. Figures 14, 15, 16 were created using the WAVOS GUI.

WAVOS has many features to facilitate analysis with the DWT and CWT, including: both command-line and graphical user interfaces; automatic processing of multiple time series simultaneously; interfaces to multiple standard file formats (including .csv,.xls, .txt, and .mat); automated detection and extraction of multiple statistics of interest (period, phase, amplitude, peak-to-trough measurements, and Rayleigh synchronization measurements); visualization tools for both exploration and analysis; utilities for smoothing, detrending, and statistical significance testing using the DWT; and multiple ridge selection algorithms, including local maximum and simulated annealing (the "crazy climber" method of [20]). Complete details are provided in the WAVOS documentation (at the download link).

Future development of WAVOS will concentrate on feature extraction from circadian signals, in particular allowing end-users to select user-defined phase markers for further analysis.

## Availability and requirements

• **Project name: **WAVOS - Wavelet Analysis and Visualization of Oscillatory Signals

• **Project home page: **http://sourceforge.net/p/wavos/home/Home/

• **Operating Systems: **Cross-platform with MATLAB 2007a or later

• **Programming language: **MATLAB

• **Other requirements: **MATLAB 2007a or later

• **License: **Modified BSD

## Competing interests

The authors declare that they have no competing interests.

## Authors' contributions

RH wrote the initial version of WAVOS under the supervision of GB. Subsequent versions were prepared under the supervision of LRP. GB wrote the edge reweighting code to address implicit periodization edge effects in the CWT. All authors contributed equally to the authorship of the manuscript. All authors read and approved the final manuscript.
